# Genome-wide DNA methylation study of hip and knee cartilage reveals embryonic organ and skeletal system morphogenesis as major pathways involved in osteoarthritis

**DOI:** 10.1186/s12891-015-0745-5

**Published:** 2015-10-09

**Authors:** Erfan Aref-Eshghi, Yuhua Zhang, Ming Liu, Patricia E. Harper, Glynn Martin, Andrew Furey, Roger Green, Guang Sun, Proton Rahman, Guangju Zhai

**Affiliations:** Discipline of Genetics, Faculty of Medicine, Memorial University of Newfoundland, St. John’s, NL Canada; Division of Orthopedics, Faculty of Medicine, Memorial University of Newfoundland, St. John’s, NL Canada; Disicpline of Medicine, Faculty of Medicine, Memorial University of Newfoundland, St. John’s, NL Canada; Department of Twin Research & Genetic Epidemiology, King’s College London, London, UK

**Keywords:** Osteoarthritis, Epigenome, DNA methylation, Skeletal morphogenesis, Hip, Knee

## Abstract

**Background:**

Evidence suggests that epigenetics plays a role in osteoarthrits (OA). The aim of the study was to describethe genome wide DNA methylation changes in hip and knee OA and identify novel genes and pathwaysinvolved in OA by comparing the DNA methylome of the hip and knee osteoarthritic cartilage tissues withthose of OA-free individuals.

**Methods:**

Cartilage samples were collected from hip or knee joint replacement patients either due to primary OA or hip fractures as controls. DNA was extracted from the collected cartilage and assayed by Illumina Infinium HumanMethylation450 BeadChip array, which allows for the analysis of >480,000 CpG sites. Student T-test was conducted for each CpG site and those sites with at least 10 % methylation difference and a *p* value <0.0005 were defined as differentially methylated regions (DMRs) for OA. A sub-analysis was also done for hip and knee OA separately. DAVID v6.7 was used for the functional annotation clustering of the DMR genes. Clustering analysis was done using multiple dimensional scaling and hierarchical clustering methods.

**Results:**

The study included 5 patients with hip OA, 6 patients with knee OA and 7 hip cartilage samples from OA-free individuals. The comparisons of hip, knee and combined hip/knee OA patients with controls resulted in 26, 72, and 103 DMRs, respectively. The comparison between hip and knee OA revealed 67 DMRs. The overall number of the sites after considering the overlaps was 239, among which 151 sites were annotated to 145 genes. One-fifth of these genes were reported in previous studies. The functional annotation clustering of the identified genes revealed clusters significantly enriched in skeletal system morphogenesis and development. The analysis revealed significant difference among OA and OA-free cartilage, but less different between hip OA and knee OA.

**Conclusions:**

We found that a number of CpG sites and genes across the genome were differentially methylated in OA patients, a remarkable portion of which seem to be involved in potential etiologic mechanisms of OA. Genes involved in skeletal developmental pathways and embryonic organ morphogenesis may be a potential area for further OA studies.

**Electronic supplementary material:**

The online version of this article (doi:10.1186/s12891-015-0745-5) contains supplementary material, which is available to authorized users.

## Background

Osteoarthritis (OA), affecting 250 million people worldwide, is the most common form of arthritis [1]. It is characterized by gradual loss of articular cartilage and subchondral bone changes, presents with joint pain, stiffness, joint deformity and disability [2], and imposes a great socio-economic burden on societies, mainly as a result of hip and/or knee involvement [3].

OA is a multifactorial condition resulting from the combination and interaction of natural and environmental factors [4]. Age, gender, obesity, previous joint injury, mal-alignments, and genetics are known to be of the major risk factors for OA; yet, the etiology of OA remains incompletely elucidated [5]. In pathology, an imbalance between catabolism and anabolism of the molecules in the cartilage extracellular matrix is a major finding in OA [6]. Since these changes are suggested to result from an altered gene expression related to epigenetic modifications of the OA candidate genes [7], it is hypothesized that epigenetic changes in chondrocytes could be a key factor of OA pathogenesis [8]. DNA methylation is by far the most extensively studied epigenetic regulator in complex diseases, and it has long been thought that its changes plays a key role in the onset and progression of complex diseases by linking the genetic and environmental risk factors [9].

To date, only few studies have been undertaken to examine the role of DNA methylation in OA. Candidate gene studies have shown the up-regulation of catabolic factors of Matrix metallopeptidase 9 (*MMP9*), Matrix metallopeptidase 13 (*MMP13*), Leptin receptor (*LEPR*), and A disintegrin and metalloproteinase with thrombospondin motifs 4 (*ADAMTS4*) [7], as well as the down-regulation of anabolic molecule collagen, type IX, alpha 1 (*COL9A1*) [10], caused by the promoter hypo- and hyper- methylations of the corresponding genes. Demethylation of an enhancer element within the nitric oxide synthase (*NOS*) gene is shown to increase transcription through elevated binding of the transcription factor NF-κB, which leads to suppression of the synthesis of cartilage matrix [11]. DNA methylation can also modulate the effect of OA genetic susceptibility loci; for instance, the effect of single nucleotide polymorphism (SNP) rs143383 in *GDF5* -- the most replicated genetic association locus in OA - is thought to be caused by the methylation level variability of the CpG dinucleotide created at the location of the SNP, leading to altered expression of the gene [12]. The handful of genome wide methylation studies performed to date have also identified several potential candidate genes including runt-related transcription factor 1&2 (*RUNX1*, *RUNX2*), transforming growth factor beta 1 (*TGFB1*), micro RNA 128 (*miR*-*128*) and collagen, type XI, alpha 2 *COL11A2* [13], suggesting the involvement of inflammation and immunity in OA pathogenesis [14]. Despite the invaluable information obtained about the pathogenesis of complex diseases from epigenetic studies, the area still remains as one of the least investigated fields in OA research.

In the present study, we conducted a genome wide DNA methylation analysis in OA-free and OA-affected cartilage from human hips and knees using the Illumina Infinium HumanMethylation450 BeadChip in the hope of providing novel insights into the pathogenesis and treatment of OA.

## Methods

### Samples and patient’s information

The study was part of the ongoing Newfoundland Osteoarthritis Study (NFOAS) that was initiated in 2011, aiming at identifying novel genetic, epigenetic, and biochemical markers for OA [15–17]. OA patients were recruited from those who underwent total knee or hip joint replacement due to primary OA between November 2011 and December 2013 in St. Clare’s Mercy Hospital and Health Science Centre General Hospital in St. John’s, the capital city of Newfoundland and Labrador (NL), Canada. OA-free controls were recruited in the same hospitals from those who underwent hemiarthroplasty of the hip due to hip fracture with no evidence of OA. OA diagnosis was made based on the American College of Rheumatology criteria [18, 19] and the judgement of the attending orthopaedic surgeons. Cartilage samples were collected from the articular surfaces of the tibial plateau or femoral head where the OA lesion occurred. The pathology report of the cartilage following the surgery was reviewed for all subjects to ensure the consistency of the diagnosis and the status of cartilage degeneration among the control subjects.

Demographic information was obtained by a self-administered questionnaire with the help of the research staff if necessary. Anthropometric data including height and weight was retrieved from their hospital admission and medical records and body mass index (BMI) was calculated by dividing weight in kilograms by squared height in meters. Age was calculated at the time of the surgery.

### DNA extraction

Four pieces (~200 mg each) of cartilage tissues were retained from either tibial plateau or femoral heads during the surgery. The samples were then flash frozen and stored in liquid nitrogen until the experiment. Up to 200 mg frozen cartilage tissue was transferred to the homogenizing cylinder together with 1 ml TRIzole lysis reagent and 200 μl guanidine thiocyanate and homogenized using a cryogenic mill (Spex Freezer Mill, model 6770, Metuchen, New Jersey, USA) with the following parameters: two cycles of 2 min grinding at maximum frequency with 10 min cooling down between grinding cycles. The homogenate was then transferred to a new 2 ml RNase free tube and incubated for 5 min at room temperature. Then, 200 μl chloroform was added and the mix was vortexed vigorously, before being incubated for 2–3 min, followed by a centrifugation at 12,000xg at −4 °C for 15 min. Following centrifugation, the sample separated into 3 phases: the aqueous phase containing RNA, the interphase, and the organic phase containing DNA. The DNA was extracted using Phenol-Chloroform method from the interphase and organic phase.

### DNA methylation profiling

DNA methylation assay was conducted using the HumanMethylation 450 Bead-Chip microarray (Illumina, San Diego, California, USA), which analyzes the methylation status of 485,000 methylation sites throughout the genome, covering 99 % of RefSeq genes at an average of 17 CpG sites per gene across the 5-UTR, gene promoter regions, first exon, gene body, and 3-UTR, and covering 96 % of University of California, Santa Cruz-defined CpG islands and their flanking regions. Briefly, DNA is first bisulfite converted, which results in unmethylated cytosines being converted to uracils, whereas methylated cytosines are not converted. The bisulfite-converted DNA is amplified, fragmented and hybridized to the arrays. For each CpG site, methylation levels are measured by probes attached to beads, one each for unmethylated and methylated DNA, followed by allele-specific base extension that includes a fluorescent label. Different labels are used for the T (unmethylated) or C (methylated) alleles. The array is fluorescently stained and scanned, and the intensities of the unmethylated and methylated bead types are measured. DNA methylation values, described as “beta values (β)”, are recorded for each locus in each sample and represent the ratio of the intensity of the methylated bead type to the combined locus intensity. The array has several features that make it a powerful option for genome-wide DNA methylation profiling: (i) multi sample format allows for interrogation of 12 samples on a single BeadChip i.e. it is high-throughout and cost-effective; (ii) low sample input (500 ng genomic DNA); (iii) reproducibility (>0.98 between technical replicates) [20, 21]. The genome-wide DNA methylation data is available at http://www.ncbi.nlm.nih.gov/geo/query/acc.cgi?acc=GSE73626 with accession number GSE73626.

### Statistical analysis

R packages minfi (version 1.6.0) [22] and minfiData (version 0.3.4) [23] was used in R version 3.1.2 to convert signal intensity data into methylation data. β values were calculated as M/(M + U), where M represents the fluorescent signal of the methylation probe and U represents the methylation signal of the unmethylated probe. The β values range from 0 (no methylation) to 1 (100 % methylation) [24].

For the purpose of quality control, CpG probes with detection *p*-value above 0.01, those located in sex chromosomes and at SNPs, and those with deviation from bimodal distribution were removed from further analysis. In order to eliminate the difference caused by two type design probes (type I and type II), beta-mixture quantile normalization (BMIQ) method [25] was used to normalize the raw methylation level data.

To identify DMRs, the average beta values were compared between the groups of interest using a student T-test assuming equal variances [26]. Given the small sample size, we were not able to correct for multiple testing as none of our tests reached the strict threshold of genome wide significance. Instead, to minimize false positives, we reported the loci with at least 10 % methylation difference and *p*-value ≤ 0.0005. The identified loci were examined using a linear regression model to determine if they were associated with age. Genomic annotation of DMRs was carried out using the Infinium HumanMethylation450 BeadChip annotation file (http://www.illumina.com/products/methylation_450_beadchip_kits.html).

### Gene ontology analysis

DAVID bioinformatics database functional tool [27] was used to identify the enriched gene ontology (GO) terms. Gene symbols were used as input for the analysis. Medium classification stringency was used. Enriched GO terms with a Bonferroni corrected *p*-value of less than 0.05 were reported.

### Phenotype clustering

Hierarchical clustering and multiple dimensional scaling were performed on all individuals after genome wide methylation distance reduction. The distance was calculated between samples taking into account the genome wide methylation levels of each sample. Then a matrix containing the pairwise distances between the samples was created. Using singular value decomposition, the matrix was transformed into three matrixes, two of which were orthogonal (U and V) and one was diagonal (D). A new matrix was generated using the multiplication of V and D, containing 12 rows representing each sample and 24 columns representing each dimension. Every two dimensions were plotted against each other. Clustering were further illustrated using heat maps of the top 800 loci with the greatest variations in methylation levels across the entire study population after the same methodology of dimension reduction. To obtain these 800 sites, the cross population variance for each CpG site was calculated, the clustering was performed for the top 200, 400, 600, 800, and above sites with the highest variance. The top 800 samples resulted in the best visual grouping of the three phenotypes while the numbers beyond this figure did not change the pattern.

### Ethics statement

The study protocol was approved by the Health Research Ethics Authority (HREA) of Newfoundland and Labrador and a written informed consent was obtained from all the participants.

## Results

### Subjects

The study included cartilage samples from 5 patients with hip OA, 6 patients with knee OA, and 7 OA-free hip controls. All subjects were females. The OA-free population were on average about 10 years older than the affected group and had a lower BMI. Table 1 shows the characteristics of the study population.

**Table 1:** Characteristics of study population

	OA-free Hip	Knee OA	*P*	Hip OA	*P*
Age	78.2 ± 11.6	65.3 ± 10.6	0.06	64.4 ± 13.8	0.09
BMI	26.0 ± 4.6	34.6 ± 8.3	0.03	32.3 ± 9.5	0.15

Figures are Mean ± standard deviation; the *p*-vales were obtained using student’s T-test between OA affected and OA-free individuals

### Differentially methylated loci

A total of 384,266 CpG sites were included in the final analysis after quality control. A total of 72, 26, and 103 CpG sites was identified from the comparison of knee OA, hip OA, and combined knee/hip OA versus hip controls, respectively. The comparison of hip and knee OA resulted in 67 CpG sites. After removing the overlaps between these analyses, a total of 239 CpG sites showed more than 10 % difference in β values among the comparison groups with all *p* <0.0005. Methylation levels of these sites were not associated with age. Almost half of the sites (53 %) showed hypo-methylation and the remainders represented hyper-methylation in OA compared to controls. This was, however, reversed among the sites with the highest methylation difference, since most of them were hypo-methylated in OA as seen in Table 2. Additional file 1: Table S1 presents the β value differences in each comparison. Among the reported sites, 151 sites were annotated to 145 genes; 119 of the sites are located in CpG islands; 79 sites are located in enhancers, 46 in regions with regulatory features, and 28 in DNAse Hypersensitivity sites. From the sites annotated to genes, the majority (46 %) were located in gene bodies, 11 % were in 5’UTR, 11 % were in 3’UTR, 5 % were in the first exon, and 27 % were located within 1500 bp upstream the transcription start site. Table 2 shows the DMRs with β value differences above 15 % between knee/hip OA and OA-free cartilage. The complete list of the DMRs is presented in the Additional file 1: Table S1.

**Table 2:** Top CpG sites differentially methylated in knee/hip OA compared to OA-free cartilage^a^

CpG	Δβ	*P*-value	Gene symbol	UCSC location group	UCSC island group	Enhancer
cg22669656	−0.22	0.0004	*PGS1*	Body		Yes
cg11905061	0.21	0.0004	*AGAP1*	Body	S_Shore	
cg27390206	−0.21	0.0002	*BLMH*	Body		Yes
cg09140531	−0.21	0.0004				Yes
cg14223856	−0.21	0.0005				Yes
cg13688786	−0.20	0.0003	*MYO18A*	Body		Yes
cg22022821	−0.20	0.0002				
cg10340048	−0.20	0.0002				Yes
cg02464866	−0.20	0.0004			N_Shore	Yes
cg05033952	−0.20	0.0001			N_Shore	
cg19629120	−0.19	0.0005	*EFCAB6*	3'UTR		
cg04973183	−0.19	0.0004				Yes
cg00150785	−0.19	0.0004			N_Shore	Yes
cg12027254	−0.19	0.00004	*TNRC6C*	Body		Yes
cg14068309	−0.19	0.0002	*EIF2B1*	3'UTR		
cg13556934	−0.18	0.0003			N_Shelf	Yes
cg14022778	−0.18	0.0004	*FHAD1*	Body		
cg02017450	0.18	0.0004				Yes
cg23074762	−0.18	0.0002	*CHSY1*	Body		Yes
cg04228742	−0.18	0.0002				
cg22203890	−0.18	0.0005				
cg17611936	0.18	0.0003	*PRKAG2*	Body		
cg07107113	−0.17	0.0004	*FBLIM1*	5'UTR	S_Shore	
cg12582728	−0.17	0.0004				Yes
cg07404223	−0.17	0.0002				Yes
cg25002179	−0.16	0.0005	*STARD13*	5'UTR;Body;TSS1500		Yes
cg17025149	−0.16	0.0001				Yes
cg26043955	−0.16	0.0004				Yes
cg26919145	−0.16	0.0002	*LDLRAD3*	Body		Yes
cg09425279	−0.16	0.0003			N_Shelf	
cg06712559	0.16	0.0004	*AGRN*	Body	Island	Yes
cg02099390	−0.16	0.0002	*OSBPL10*	Body		Yes
cg11805414	−0.16	0.0005				Yes
cg04038680	−0.16	0.0002	*SHISA9*	Body		Yes
cg25341923	−0.15	0.0001	*KRTAP4-7*	TSS1500		
cg14728071	−0.15	0.00004	*MLLT10*	3'UTR		
cg03667871	−0.15	0.0004	*NEK7*	TSS1500	N_Shore	
cg13258453	−0.15	0.0002				Yes
cg23010507	−0.15	0.0001			S_Shelf	Yes
cg12158488	−0.15	0.0002				Yes

^a^Δβ: difference in methylation value between sample groups (OA cartilage - intact); UCSC: University of California, Santa Cruz; 5’-UTR: 5’-untranslated region; N: North; S: South; TSS 200: within 200 bp of transcription start site

### Functional annotation clustering of the differentially methylated loci

The annotation clustering was conducted for the 145 genes. Table 3 shows those GO terms with a Bonferroni corrected *p*-value < 0.05. The most significant terms are related to skeletal and embryonic organ system development and homeobox (HOX). By increasing the classification stringency, the GO terms were classified into 31 clusters, among which only two yielded significant Bonferroni corrected *p*-values including Embryoinic and skeletal system development and HOX genes (enrichment scores 6.48 and 5.52, respectively). The analysis was repeated after the removal of 33 genes which were only identified in the comparison of knee OA and hip OA. Similar to previous clustering, the GO terms included skeletal and embryonic system development, but no HOX genes.

**Table 3:** Enrichment clustering of the differentially methylated genes^a^

Term	Gene count	% of the total genes entered	Genes in each pathway from our result	Fold Enrichment	Bonferroni *P*-value
GO:0048705 ~ skeletal system morphogenesis	14	9.6	*GSC, TBX15, PAX1, GLI3, HOXB3, HOXD9, HOXC8, HOXD8, HOXC9, OSR2, HOXD3, HOXB6, ALX4, RUNX2*	14.96	8.39E-09
GO:0048704 ~ embryonic skeletal system morphogenesis	10	6.9	*HOXD9, HOXB3, GSC, OSR2, TBX15, HOXC9, HOXD3, HOXB6, ALX4, GLI3*	21.00	9.89E-07
GO:0001501 ~ skeletal system development	17	11.7	*CYP24A1, GSC, TBX15, GLI3, PAX1, GLI1, HOXD9, HOXB3, HDAC4, HOXC8, HOXD8, HOXC9, OSR2, HOXD3, HOXB6, ALX4, RUNX2*	6.38	8.98E-06
GO:0048562 ~ embryonic organ morphogenesis	12	8.3	*HOXD9, HOXB3, GSC, OSR2, TBX15, HOXC9, HOXD3, HOXB6, VAX2, ALX4, GLI3, GLI1*	10.80	1.43E-05
GO:0048706 ~ embryonic skeletal system development	10	6.8	*HOXD9, HOXB3, GSC, OSR2, TBX15, HOXC9, HOXD3, HOXB6, ALX4, GLI3*	15.54	1.55E-05
GO:0003002 ~ regionalization	13	8.9	*GSC, VAX2, PAX1, GLI3, GLI1, HOXB3, HOXD9, HOXC8, HOXC9, HOXD8, HOXD3, HOXB6, ALX4*	7.91	9.34E-05
GO:0043565 ~ sequence-specific DNA binding^b^	20	13.8	*GSC, ESRRG, VAX2, MEIS1, GLI3, GLI1, HOXD9, HOXB3, HDAC4, HOXC8, HOXD8, MEIS2, HOXC9, HAND2, GATA6, HOXD3, HOXB6, THAP1, ALX4, ETV6*	4.03	1.02E-04
GO:0048568 ~ embryonic organ development	12	8.3	*HOXD9, HOXB3, GSC, OSR2, TBX15, HOXC9, HOXD3, HOXB6, VAX2, ALX4, GLI3, GLI1*	8.35	2.05E-04
Homeobox^b^	12	8.3	*HOXD9, HOXB3, HOXC8, GSC, HOXD8, MEIS2, HOXC9, HOXD3, HOXB6, VAX2, ALX4, MEIS1*	6.96	3.14E-04
GO:0007389 ~ pattern specification process	14	9.6	*GSC, VAX2, PAX1, GLI3, GLI1, HOXB3, SEMA5A, HOXD9, HOXC8, HOXD8, HOXC9, HOXD3, HOXB6, ALX4*	6.27	3.57E-04
SM00389:HOX^b^	12	8.3	*HOXD9, HOXB3, HOXC8, GSC, HOXD8, MEIS2, HOXC9, HOXD3, HOXB6, VAX2, ALX4, MEIS1*	5.65	5.84E-04
IPR017970:Homeobox, conserved site^b^	12	8.3	*HOXD9, HOXB3, HOXC8, GSC, HOXD8, MEIS2, HOXC9, HOXD3, HOXB6, VAX2, ALX4, MEIS1*	6.62	6.07E-04
IPR001356:Homeobox^b^	12	8.3	*HOXD9, HOXB3, HOXC8, GSC, HOXD8, MEIS2, HOXC9, HOXD3, HOXB6, VAX2, ALX4, MEIS1*	6.54	6.87E-04
IPR012287:Homeodomain-related^b^	12	8.3	*HOXD9, HOXB3, HOXC8, GSC, HOXD8, MEIS2, HOXC9, HOXD3, HOXB6, VAX2, ALX4, MEIS1*	6.46	7.77E-04
GO:0009952 ~ anterior/posterior pattern formation^b^	10	6.9	*HOXD9, HOXB3, HOXC8, HOXD8, HOXC9, HOXD3, HOXB6, ALX4, PAX1, GLI3*	8.55	0.002
DNA-binding region:Homeobox^b^	10	6.9	*HOXD9, HOXB3, HOXC8, GSC, HOXD8, HOXC9, HOXD3, HOXB6, VAX2, ALX4*	7.34	0.004
IPR001827:Homeobox protein, antennapedia type, conserved site^b^	5	3.4	*HOXB3, HOXC8, HOXD8, HOXD3, HOXB6*	26.69	0.01
GO:0043009 ~ chordate embryonic development^b^	13	8.9	*GSC, TBX15, PAX1, GLI3, HOXB3, HOXD9, HOXC9, OSR2, HAND2, GATA6, HOXD3, HOXB6, ALX4*	4.70	0.02
GO:0009792 ~ embryonic development ending in birth or egg hatching^b^	13	8.9	*GSC, TBX15, PAX1, GLI3, HOXB3, HOXD9, HOXC9, OSR2, HAND2, GATA6, HOXD3, HOXB6, ALX4*	4.66	0.02
GO:0003700 ~ transcription factor activity^b^	21	14.5	*TBX15, GSC, ESRRG, VAX2, MEIS1, GLI3, GLI1, HOXD9, HOXB3, HOXC8, HOXD8, MEIS2, HOXC9, HAND2, GATA6, HOXD3, MLLT10, HOXB6, ALX4, ETV6, RUNX2*	2.64	0.02
Developmental protein^b^	17	11.7	*GSC, ZNF521, VAX2, MEIS1, PAX1, GLI1, HOXD9, HOXB3, SEMA5A, HOXC8, HOXD8, HOXC9, HAND2, HOXD3, HOXB6, ROBO2, ALX4*	3.06	0.03

^a^All of the GO terms above were clustered into one annotation cluster with an overall enrichment score of 3.95. ^b^The GO terms were only significant before the removal of the genes differentially methylated between hip OA and knee OA

### Clustering of hip OA, knee OA and OA-free cartilage

**Fig. 1:**
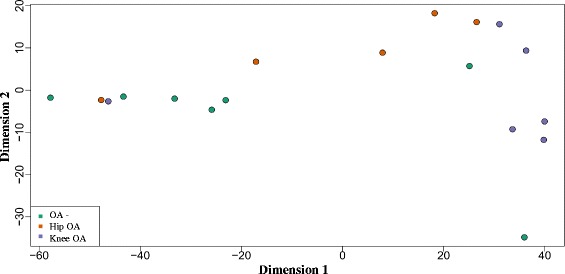
Multiple dimensions scaling of hip OA, knee OA, and OA-free hip cartilage. *Similarities between hip OA, knee OA, and OA-free cartilage, drawn from log-spectral decompositions for each subject as represented in the two-dimensional space by multiple dimensional scaling (MDS). Each dot represents one sample. Colors represent the type of involvement and the site samples obtained. X- and Y- axes represent the first and the second dimension reductions

**Fig. 2:**
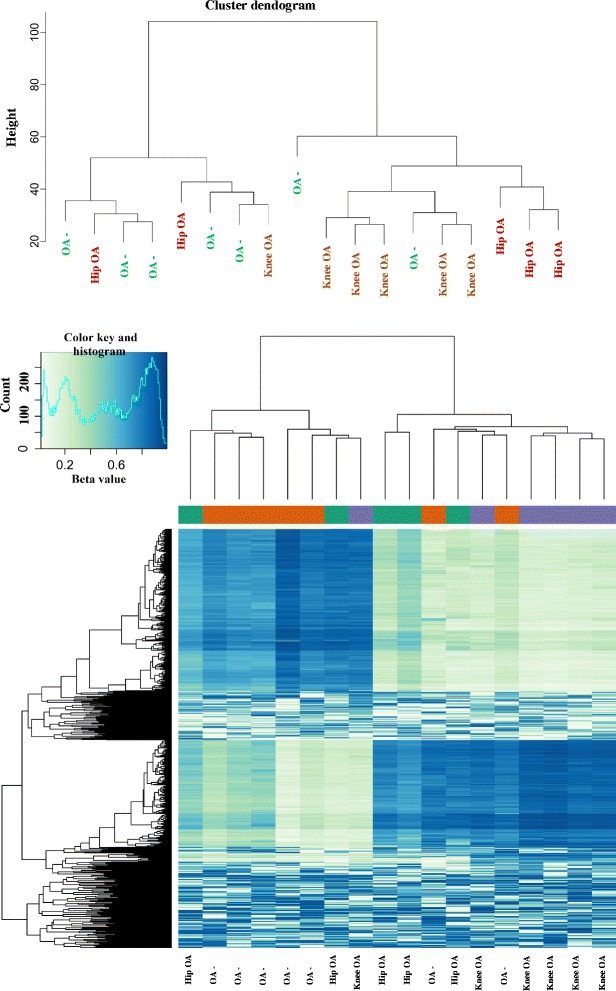
Hierarchical clustering and heat map of hip OA, knee OA, and OA-free controls*. *Top: Cluster dendogram was created using the genome wide information; Bottom: Heat map shows the top 800 CpG sites with the most variation across hip OA, knee OA, and OA-free hip cartilage samples. Rows represent CpG sites. Columns represent samples. Dark blue indicates hypermethylation and light blue/white indicates hypomethylation

We used multiple dimensional scaling and hierarchical clustering to classify the three phenotypes in the study, i.e. hip OA, knee OA, and OA-free hip cartilage. Due to the small sample size, the classification was not perfect; however, some trends were observed which are worthy of consideration. As it is seen in the plots (Fig. 1), samples from each phenotype tend to cluster together, although a few outliers exist. Overall, OA-free hip cartilage samples tend to be different from hip OA and knee OA samples. Although hip OA and knee OA samples are very close together, the similarity to OA-free hip cartilage is more seen in hip OA rather than knee OA samples. Scaling beyond the 2^nd^ dimension was not informative (not shown). Similar patterns are observed from the heat map and dendogram as shown in Fig. 2.

## Discussion

Our study is one of the few reports on the status of genome wide methylation of DNA from OA-free and OA affected human cartilage. We found a number of CpG sites differentially methylated in hip and knee OA, identified the pathways enriched in the sites, and attempted to classify hip and knee OA and OA-free cartilage according to their genome wide DNA methylation profiling.

The majority of the CpG sites we identified were novel and only about one fifth of them were reported by the previous epigenetic studies of OA (Additional file 1: Table S2) [13, 14, 28, 29]. Similarly, most of the genes differentially methylated were not known to play a role in OA, although several of them were previously reported as candidate genes to OA or other bone metabolic conditions. They include cg07902192 in *RUNX2*, involved in the regulation of matrix metallopeptidase 13 in OA cartilage [30], cg03050981 in *LEPR*, associated with knee OA [31], bone marrow density and bone hemostasis [32], cg05516020 in *CLCN7*, associated with bone marrow density [33], cg17279365 in *ESRRG*, associated with multiple bone disease phenotypes [34], cg14340103 in *IL21*, being upregulated in synovial biopsies of rheumatoid arthritis patients [35], cg18637380 in *MTHFD1*, associated with response to osteosarcoma chemotherapy [36], cg10629004 in *PAX1*, associated with congenital scoliosis [37], and cg10908116 in the alpha-1 subunit of collagen type IV gene (*COL4A1*).

Although most of the DMR genes in our study were of unknown significance in OA, the functional analysis revealed their enrichment in relevant pathways; i.e. skeletal and embryonic organ system development and homeobox. The latter was only found from the genes differentially methylated in the comparison of knee OA and hip OA methylation. Since homeobox genes are responsible for the body segmentation procedure and specification of lower limbs from upper limbs, it is likely that the DMRs from knee OA and hip OA comparison represent the processes required for body segment specifications rather than the underlying genetic difference between hip OA and knee OA. The same gene ontology term has also been reported by Den Hollander et al. who made a comparison between genome wide methylation of articular cartilage DNA from hip OA and knee OA [28]. The small number of genome wide methylation studies of OA have reported inflammation and immunity [14, 29], transforming growth factor beta signalling [13], and KEGG and developmental pathways [38]. Our study, however, points out the involvement of skeletal system development in OA , which is in accordance with findings of Delgado-Calle J et al. who reported the enrichment of genes associated with the development of the appendicular skeleton and limb morphogenesis in a genome wide methylation study of femoral bone [39]. In this process the anatomical and physical structures of the skeleton are generated and organized. Skeletal shape, which is tightly regulated by genetics [40], is suggested as a possible mechanism for the influence of genetics in OA incidence. An abnormal center-edge angle and acetabular dysplasia are shown to be associated with an increased risk of hip OA [41], and a significant difference in the shape of the intercondylar notch between the OA and non-OA individuals is reported [42]. Wnt signalling and bone morphogenetic proteins are among the pathways involved in OA which also control skeletal development in animal models, and it is suspected that their mechanism of action in OA could be due to their effect on skeletal shape [43].

Our clustering analysis of the three phenotypes in the study clearly shows that OA-affected cartilage has a distinct methylation profiling compared to OA-free cartilage. The hip OA and knee OA clustering, however, is not perfect and is suggesting that although hip OA and knee OA could have different epigenomic landscape, they are very similar to each other. The only minor differences observed in hip OA and knee OA might only be joint specific differences that could have been observed if OA-free knee and hip cartilage were studied. The only comparison of the hip OA and knee OA was done by Den Hollander et al. [37] who successfully grouped hip OA and knee OA into separate clusters. The main conclusion in that study was based on the OA-affected cartilage, and similar to our study the major pathways they identified enriched in the DMRs between hip OA and knee OA, was homeobox, which strengthens the hypothesis that the observed differences in hip and knee OA might be due to differences in the joints rather than the disease status.

Our study is limited by several factors. Due to technical issues we studied a small sample size, and as the result, none of the DMRs reported reached Bonferroni corrected significance. The cases and controls were different in age, which might influence the status of DNA methylation. The controls were selected from the population of patients with possible osteoporosis who may not necessarily represent healthy cartilage epigenome. In addition, we did not have information on the pathological scoring of the OA joints, which could partially explain why the clustering was not perfect. Due to financial issues we did not validate the methylation levels using alternative methods such as pyrosequencing and did not perform functional experiments to add more to the mechanism of involvement. These limitations will likely tackled by future studies attempting to replicate and further studying our findings. Despite these limitations, the trends observed in the study were informative and add to the current knowledge on the pathogenesis of OA.

## Conclusion

Through a genome wide methylation study of OA-free and OA-affected human cartilage, we were able to identify a number of CpG sites with methylation changes in OA. We also reported that genes involved in skeletal system morphogenesis are differentially methylated in OA and might be candidate genes for further OA studies. We found a small difference between the overall landscapes of hip OA and knee OA; however, OA-free hip cartilage samples were significantly different from the OA-affected ones. Our findings shed light on the pathophysiology of OA and can pave the road for further research in the field.

## Additional file

Additional file 1: Table S1. CpG sites differentially methylated in knee/hip OA compared to healthy cartilage*. **Table 2**. The genes and CpG sites commonly reported between our study and previous epigenome-wide studies of OA. (DOCX 36 kb)

## Abbreviations

OA: Osteoarthritis
ADAMTS4: A disintegrin and metalloproteinase with thrombospondin motifs 4
COL9A1: Anabolic molecule collagen, type IX, alpha 1
NOS: Nitric oxide synthase
SNP: Single nucleotide polymorphism
RUNX1: RUNX2, Runt-related transcription factor 1&2
TGFB1: Transforming growth factor beta 1
miR-128: Micro RNA 128
COL11A2: Collagen, type XI, alpha 2
NFOAS: Newfoundland Osteoarthritis Study
NL: Newfoundland and Labrador
BMI: Body mass index
GO: Gene ontology
HOX: Homeobox
COL4A1: Alpha-1 subunit of collagen type IV gene
