# Supplementary material for: Genome-wide DNA methylation study of hip and knee cartilage reveals embryonic organ and skeletal system morphogenesis as major pathways involved in osteoarthritis
Source: BMC Musculoskelet Disord. 2015 Oct 9;16:287. doi: 10.1186/s12891-015-0745-5 (PMC4600269; doi:10.1186/s12891-015-0745-5)
Supplement: Additional file 1: Table S1. — CpG sites differentially methylated in knee/hip OA compared to healthy cartilage*. Table 2. The genes and CpG sites commonly reported between our study and previous epigenome-wide studies of OA. (DOCX 36 kb) [file 12891_2015_745_MOESM1_ESM.docx]

Additional file 1

Table S1- CpG sites differentially methylated in knee/hip OA compared to healthy cartilage‎*

| **CpG** | **Gene** | **Region** | **Difference in Beta values** | | | |
| --- | --- | --- | --- | --- | --- | --- |
|  |  |  | **Hip/knee OA vs. OA free cartilage** | **Knee OA vs. OA free ‎cartilage** | **Hip OA vs. OA free ‎cartilage** | **Hip OA vs. Knee OA** |
| cg27394794 | *A1CF* | Body | -0.103021 |  |  |  |
| cg04573661 | *AASDH* | TSS1500 |  | -0.205279 |  |  |
| cg03213833 | *ADARB2* | Body |  | -0.286976 |  |  |
| cg11905061 | *AGAP1* | Body | 0.210268 |  |  |  |
| cg06712559 | *AGRN* | Body | 0.159346 |  |  |  |
| cg12899423 | *ALX4* | Body | 0.147695 | 0.182835 |  |  |
| cg19894728 | *ATXN7L1* | Body | -0.114155 |  |  |  |
| cg23730617 | *B3GALNT1* | TSS1500 |  |  |  | -0.185146 |
| cg25764534 | *BIN3* | Body |  | 0.104192 |  |  |
| cg27390206 | *BLMH* | Body | -0.209088 |  |  |  |
| cg27143664 | *C14orf38* | Body | -0.130948 |  |  |  |
| cg23276912 | *C1orf212* | TSS1500 |  | -0.113101 |  |  |
| cg10916651 | *C1QA* | TSS200 |  |  |  | 0.108015 |
| cg08166362 | *C3orf37* | Body |  |  |  | -0.112695 |
| cg22199118 | *C8orf34* | 5'UTR;1stExon | -0.104984 |  |  |  |
| cg13551505 | *CACNA2D4* | Body | -0.101028 | -0.112074 |  |  |
| cg23327859 | *CCNT1* | TSS1500 |  |  |  | -0.117283 |
| cg27106290 | *CDH12* | TSS200 |  | -0.182634 |  |  |
| cg10303842 | *CDH12* | 5'UTR;1stExon |  | -0.105454 |  |  |
| cg20429981 | *CDK11B;LOC728661;CDK11B* | Body;5'UTR;5'UTR |  |  |  | 0.132189 |
| cg23074762 | *CHSY1* | Body | -0.181762 |  |  |  |
| cg01413281 | *CHUK* | TSS1500 |  | -0.210765 |  |  |
| cg05516020 | *CLCN7* | Body | -0.120175 |  |  |  |
| cg26066361 | *CLEC7A* | 1stExon |  |  |  | 0.111099 |
| cg06551161 | *CLP1;CLP1* | TSS1500 |  |  |  | -0.218961 |
| cg12138483 | *CLPP* | TSS1500 | -0.132099 |  |  |  |
| cg01487542 | *CMAH* | TSS200;Body |  |  |  | 0.136506 |
| cg10908116 | *COL4A1* | Body |  |  | -0.188257 |  |
| cg16524108 | *COQ6;FAM161B;COQ6* | TSS1500;Body;TSS1500 |  | -0.135545 |  |  |
| cg27318087 | *CPPED1* | Body |  | -0.16637 |  |  |
| cg17641876 | *CPT1B;CHKB* | TSS1500;Body |  |  | -0.1261 |  |
| cg27410679 | *CRHR1* | Body |  |  |  | 0.139596 |
| cg01462727 | *CYP24A1* | Body |  | -0.308772 |  |  |
| cg07054208 | *DCDC2* | TSS1500 |  |  |  | -0.134915 |
| cg19931902 | *DEFB129* | TSS1500 | -0.123362 |  |  |  |
| cg20728490 | *DNTT* | 5'UTR;1stExon | -0.109343 | -0.129115 |  |  |
| cg11969108 | *DPP6* | Body | -0.125924 |  |  |  |
| cg19629120 | *EFCAB* | 3'UTR;3'UTR | -0.194821 |  |  |  |
| cg14068309 | *EIF2B1* | 3'UTR | -0.185456 |  |  |  |
| cg16324018 | *ELMOD3; RETSAT* | TSS1500;Body |  | -0.10651 |  |  |
| cg14671809 | *ERC2* | 3'UTR |  | 0.21432 |  |  |
| cg17279365 | *ESRRG* | 5'UTR |  |  |  | 0.54009 |
| cg22357390 | *ETV6* | Body | -0.106365 |  |  |  |
| cg11562401 | *FAM19A5* | Body |  | -0.140515 |  |  |
| cg07107113 | *FBLIM1* | 5'UTR | -0.172251 |  |  |  |
| cg14022778 | *FHAD1* | Body | -0.182901 |  |  |  |
| cg04988917 | *FKSG83* | TSS1500 |  | -0.136044 |  |  |
| cg19177125 | *GATA6* | Body |  |  |  | -0.158489 |
| cg06679990 | *GLI1* | Body | -0.125178 |  |  |  |
| cg00362680 | *GLI3* | Body | -0.109003 |  |  |  |
| cg20148127 | *GLIS1* | 5'UTR |  | -0.131564 |  |  |
| cg17154975 | *GPR133* | Body | -0.131627 |  |  |  |
| cg07785447 | *GSC* | Body |  |  |  | 0.143192 |
| cg01163842 | *GSC* | Body |  |  |  | 0.227057 |
| cg04387592 | *GUCA1A* | 5'UTR | -0.125863 |  | -0.141964 |  |
| cg24974365 | *HAND2* | Body |  |  |  | 0.345116 |
| cg26729101 | *HBQ1* | TSS1500 |  | -0.158386 |  |  |
| cg26495711 | *HDAC4* | Body | -0.13355 |  |  |  |
| cg04625975 | *HNRNPA3P1* | Body |  |  |  | 0.110269 |
| cg01593673 | *HOXB3* | 5'UTR |  | -0.147136 |  |  |
| cg22660542 | *HOXC8* | TSS1500 |  |  |  | 0.168632 |
| cg23685155 | *HOXC9* | Body |  |  |  | 0.284029 |
| cg02773086 | *HOXD3* | TSS1500 |  | 0.30235 |  | 0.344425 |
| cg01293179 | *HOXD8* | Body |  |  |  | -0.219926 |
| cg15991405 | *HOXD9* | Body |  | -0.135009 |  | -0.228901 |
| cg12969193 | *HOXD9* | Body |  |  |  | -0.245435 |
| cg06150772 | *HRNBP3* | 5'UTR | -0.122901 | -0.140676 |  |  |
| cg19442493 | *HRNBP3* | 5'UTR |  |  | -0.101717 |  |
| cg19815720 | *HTR3C* | TSS200 | -0.121236 | -0.103268 |  |  |
| cg27027427 | *IFIT3;IFIT3* | Body;TSS1500 |  |  | -0.134842 |  |
| cg14340103 | *IL21* | TSS1500 |  |  | -0.107352 |  |
| cg15152331 | *ITGB6* | Body |  | 0.107484 |  |  |
| cg18942298 | *JPH2* | Body | -0.144872 |  |  |  |
| cg01575590 | *KCNQ2* | Body | -0.121138 |  |  |  |
| cg17022362 | *KCNQ2* | Body | -0.136883 |  |  |  |
| cg25341923 | *KRTAP4-7* | TSS1500 | -0.152813 |  |  |  |
| cg20634798 | *LCE3A* | 1stExon |  |  |  | 0.201253 |
| cg26135325 | *LCE3A* | 1stExon |  |  |  | 0.196904 |
| cg26919145 | *LDLRAD3* | Body | -0.163533 |  |  |  |
| cg03050981 | *LEPR* | 5'UTR | -0.136643 | -0.155669 |  |  |
| cg25788513 | *LOC100188947* | Body |  | -0.103142 |  |  |
| cg23343309 | *LOC150185* | TSS1500 |  | -0.116973 |  |  |
| cg07676709 | *LOC404266;HOXB6* | Body |  | -0.13161 |  |  |
| cg16770054 | *MAD1L1* | Body |  |  |  | 0.102319 |
| cg26537478 | *MEIS1* | Body |  |  |  | 0.442717 |
| cg05877497 | *MEIS1* | Body |  |  |  | 0.537868 |
| cg11362604 | *MEIS2* | Body |  |  |  | 0.286791 |
| cg03951374 | *MEIS2* | 1stExon;Body;5'UTR |  |  |  | 0.101942 |
| cg14728071 | *MLLT10* | 3'UTR | -0.152616 |  |  |  |
| cg18637380 | *MTHFD1* | TSS1500 | -0.128176 |  |  |  |
| cg13688786 | *MYO18A* | Body | -0.20458 |  |  |  |
| cg00729885 | *NBEA* | Body |  | 0.36108 |  |  |
| cg03667871 | *NEK7* | TSS1500 | -0.152465 | -0.16889 |  |  |
| cg12044531 | *NIN* | 3'UTR | -0.149347 |  |  |  |
| cg26489750 | *NLRP2* | 3'UTR | -0.123049 |  |  |  |
| cg24307499 | *NLRP2* | Body |  | -0.123286 |  |  |
| cg07429087 | *NMUR2* | 3'UTR |  | -0.104922 |  |  |
| cg12986700 | *NPFFR2* | Body;5'UTR;Body |  |  |  | 0.210622 |
| cg16170380 | *OR11A1* | 1stExon | -0.11028 |  |  |  |
| cg12080717 | *OR11L1* | 1stExon | -0.103754 |  |  |  |
| cg20867746 | *OR51S1* | TSS1500 | -0.105627 |  |  |  |
| cg02099390 | *OSBPL10* | Body | -0.159326 |  |  |  |
| cg05986505 | *OSR2* | TSS1500 |  | 0.264363 |  |  |
| cg27319188 | *PACS1* | Body |  |  |  | 0.183381 |
| cg05477457 | *PALLD* | Body |  | 0.154197 |  |  |
| cg08189448 | *PAPPA* | TSS200 |  |  |  | 0.189692 |
| cg04462132 | *PARK2* | Body |  |  | -0.120193 |  |
| cg10629004 | *PAX1* | 3'UTR |  |  | 0.212762 |  |
| cg11164441 | *PDE6B* | Body |  |  | -0.105268 |  |
| cg11320144 | *PDYN* | TSS1500 |  | -0.105703 |  |  |
| cg23975251 | *PEX14* | Body |  |  | -0.149433 |  |
| cg03068346 | *PFKP* | Body |  |  | -0.109451 |  |
| cg22669656 | *PGS1* | Body | -0.220716 |  |  |  |
| cg03803861 | *PLCXD3* | TSS1500 | 0.114606 |  |  |  |
| cg09050331 | *PRDM14* | TSS1500 |  | 0.145683 |  |  |
| cg17611936 | *PRKAG2* | Body | 0.176367 | 0.220172 |  |  |
| cg12304937 | *PRKAR1B* | Body | -0.115834 |  |  |  |
| cg16197388 | *PSG3* | 3'UTR | -0.1227 | -0.142669 |  |  |
| cg19741675 | *PSORS1C1* | 5'UTR |  |  | -0.102053 |  |
| cg01394116 | *RBM22* | TSS1500 |  | -0.164379 |  |  |
| cg21090457 | *ROBO2* | Body | -0.149873 | -0.151843 |  |  |
| cg00424286 | *RPS6KA2* | Body |  |  |  | 0.104957 |
| cg07902192 | *RUNX2* | Body | -0.143582 |  |  |  |
| cg00442802 | *SAA3P* | TSS1500 | -0.144992 |  |  |  |
| cg09699193 | *SEMA5A* | 5'UTR |  | -0.161465 |  |  |
| cg04038680 | *SHISA9* | Body | -0.155409 |  |  |  |
| cg18074184 | *SLC10A4* | TSS1500 |  |  |  | -0.13289 |
| cg14995160 | *SLC18A2* | 3'UTR |  |  | -0.103794 |  |
| cg20388916 | *SLCO2A1* | Body |  | 0.121495 |  |  |
| cg26059632 | *SPRR2A* | TSS1500 | -0.118555 |  |  |  |
| cg10661163 | *ST7OT4;ST7OT1;ST7* | TSS1500;Body;TSS1500 |  |  |  | -0.100255 |
| cg25002179 | *STARD13* | 5'UTR;Body;TSS1500 | -0.1638 |  |  |  |
| cg18847227 | *SUMF1* | TSS1500 |  |  |  | -0.132954 |
| cg23083424 | *SYNPO2L* | TSS200 | -0.119664 |  |  |  |
| cg22378919 | *TBX15* | 5'UTR |  |  |  | 0.155047 |
| cg06884495 | *TDRD9* | TSS200 |  |  |  | -0.121617 |
| cg02937313 | *THAP1* | TSS1500 |  | -0.168846 |  |  |
| cg13314965 | *TM4SF19* | TSS1500 | -0.134876 |  |  |  |
| cg12027254 | *TNRC6C* | Body | -0.185722 |  |  |  |
| cg11559731 | *TOX* | TSS1500 |  |  | 0.10719 |  |
| cg20071744 | *TRAPPC9* | Body |  |  |  | 0.110648 |
| cg16422492 | *TRIM72;PYDC1* | Body;TSS200 |  | -0.102686 |  |  |
| cg22081905 | *TRPM5* | Body |  |  |  | 0.104541 |
| cg11937920 | *UACA* | Body;TSS1500 | -0.149382 | -0.160089 |  |  |
| cg18889780 | *UCHL1* | TSS1500 |  | 0.109206 |  |  |
| cg09305680 | *UTP23* | TSS1500 |  |  |  | 0.270613 |
| cg19907796 | *VAX2* | Body | 0.126749 |  | 0.146154 |  |
| cg02774935 | *YPEL1* | TSS1500 | 0.117408 |  |  |  |
| cg06545761 | *ZCCHC14* | 3'UTR |  |  | -0.135392 |  |
| cg16727006 | *ZCCHC14* | Body |  |  | -0.142668 |  |
| cg23238231 | *ZNF521* | Body |  |  |  | -0.215518 |
| cg00150785 |  |  | -0.18856 |  |  |  |
| cg01478628 |  |  | -0.124007 |  |  |  |
| cg01549315 |  |  | 0.141595 | 0.159916 |  |  |
| cg02017450 |  |  | 0.182854 |  |  |  |
| cg02097429 |  |  | -0.115893 | -0.120603 |  |  |
| cg02464866 |  |  | -0.201143 |  |  |  |
| cg02555944 |  |  | -0.100254 |  |  |  |
| cg04228742 |  |  | -0.181463 |  |  |  |
| cg04973183 |  |  | -0.194445 | -0.218185 |  |  |
| cg05033952 |  |  | -0.200003 |  |  |  |
| cg06410273 |  |  | -0.147566 |  |  |  |
| cg07404223 |  |  | -0.166132 |  |  |  |
| cg08189043 |  |  | -0.101672 |  |  |  |
| cg08236537 |  |  | -0.129884 |  |  |  |
| cg09140531 |  |  | -0.208274 |  |  |  |
| cg09318857 |  |  | -0.103781 |  |  |  |
| cg09425279 |  |  | -0.163148 |  |  |  |
| cg09932730 |  |  | -0.112782 | -0.133961 |  |  |
| cg10201735 |  |  | -0.108855 |  |  |  |
| cg10340048 |  |  | -0.203169 |  |  |  |
| cg11351841 |  |  | -0.10315 |  |  |  |
| cg11805414 |  |  | -0.157269 |  |  |  |
| cg12158488 |  |  | -0.151087 |  |  |  |
| cg12399687 |  |  | -0.133118 |  | -0.144144 |  |
| cg12582728 |  |  | -0.167141 |  |  |  |
| cg12760319 |  |  | -0.120773 | -0.154492 |  |  |
| cg13258453 |  |  | -0.152225 |  | -0.199005 |  |
| cg13397166 |  |  | -0.131556 |  |  |  |
| cg13556934 |  |  | -0.183866 |  |  |  |
| cg13607993 |  |  | -0.110454 |  |  |  |
| cg14223856 |  |  | -0.208172 |  |  |  |
| cg15272641 |  |  | -0.1204 |  |  |  |
| cg15609373 |  |  | -0.146004 |  |  |  |
| cg15994519 |  |  | -0.108552 |  |  |  |
| cg17025149 |  |  | -0.163611 | -0.190251 |  |  |
| cg21249771 |  |  | -0.138605 |  |  |  |
| cg22022821 |  |  | -0.204191 |  | -0.265695 |  |
| cg22203890 |  |  | -0.176764 |  |  |  |
| cg23010507 |  |  | -0.151239 |  |  |  |
| cg23153661 |  |  | -0.124432 |  |  |  |
| cg24757553 |  |  | 0.12996 |  |  |  |
| cg26043955 |  |  | -0.163536 |  | -0.202862 |  |
| cg26196162 |  |  | -0.129839 |  |  |  |
| cg14592399 |  |  |  | 0.109712 |  |  |
| cg24138867 |  |  |  | -0.169935 |  |  |
| cg25843866 |  |  |  | 0.16252 |  |  |
| cg17171786 |  |  |  | 0.140878 |  |  |
| cg21776682 |  |  |  | 0.113997 |  |  |
| cg24415066 |  |  |  | 0.378201 |  |  |
| cg12754260 |  |  |  | -0.140132 |  | -0.152867 |
| cg16138557 |  |  |  | -0.131028 |  |  |
| cg13441058 |  |  |  | 0.134108 |  |  |
| cg07536144 |  |  |  | 0.163068 |  |  |
| cg08018143 |  |  |  | -0.186449 |  |  |
| cg15372218 |  |  |  | -0.12534 |  |  |
| cg23348270 |  |  |  | 0.277439 |  | 0.304979 |
| cg11246774 |  |  |  | -0.184875 |  |  |
| cg09817024 |  |  |  | 0.175003 |  | 0.175612 |
| cg14356225 |  |  |  | -0.351866 |  |  |
| cg08873424 |  |  |  | 0.167794 |  |  |
| cg15275309 |  |  |  | -0.110718 |  | -0.1184 |
| cg00333870 |  |  |  | -0.1351 |  |  |
| cg04234597 |  |  |  |  | -0.115774 |  |
| cg05334656 |  |  |  |  | 0.226141 |  |
| cg05573844 |  |  |  |  | 0.142033 |  |
| cg05552543 |  |  |  |  | -0.117198 |  |
| cg27403071 |  |  |  |  | -0.108052 |  |
| cg04588138 |  |  |  |  |  | 0.100713 |
| cg10481584 |  |  |  |  |  | 0.104582 |
| cg26469220 |  |  |  |  |  | -0.143677 |
| cg03913423 |  |  |  |  |  | -0.14046 |
| cg04517282 |  |  |  |  |  | 0.179701 |
| cg22855900 |  |  |  |  |  | 0.143314 |
| cg14671000 |  |  |  |  |  | -0.151115 |
| cg01519253 |  |  |  |  |  | 0.208831 |
| cg01419670 |  |  |  |  |  | -0.149344 |
| cg09889228 |  |  |  |  |  | -0.119094 |
| cg10111328 |  |  |  |  |  | -0.259716 |
| cg12547939 |  |  |  |  |  | 0.100249 |
| cg14132364 |  |  |  |  |  | 0.173478 |
| cg26399903 |  |  |  |  |  | 0.120465 |
| cg02622133 |  |  |  |  |  | 0.105884 |
| cg25941985 |  |  |  |  |  | -0.28141 |
| cg10536898 |  |  |  |  |  | -0.238791 |
| cg01637125 |  |  |  |  |  | 0.128925 |
| cg02166394 |  |  |  |  |  | 0.250803 |
| cg03787282 |  |  |  |  |  | 0.118125 |
| cg19506686 |  |  |  |  |  | -0.214439 |

*All p-values≤0.0005

Table S2- The genes and CpG sites commonly reported between our study and previous epigenome-wide studies of OA

| cpg | UCSC reference gene name |
| --- | --- |
| cg05877497 | *MEIS1* |
| cg11362604 | *MEIS2* |
| cg09305680 | *UTP23* |
| cg24974365 | *HAND2* |
| cg23685155 | *HOXC9* |
| cg23348270 |  |
| cg00729885 | *NBEA* |
| cg24415066 |  |
| cg02773086 | *HOXD3* |
| cg22378919 | *TBX15* |
| cg01163842 | *GSC* |
| cg04625975 | *HNRNPA3P1* |
| cg02464866 |  |
| cg22199118 | *C8orf34* |
| cg07785447 | *GSC* |
| cg05334656 |  |
| cg03913423 |  |
| cg07676709 | *LOC404266; HOXB6* |
| cg01462727 | *CYP24A1* |
| cg13556934 |  |
| cg14671000 |  |
| cg23238231 | *ZNF521* |
| cg10629004 | *PAX1* |
| cg05573844 |  |
| cg11562401 | *FAM19A5* |
| cg09889228 |  |
| cg06551161 | *CLP1* |
| cg27106290 | *CDH12* |
| cg03213833 | *ADARB2* |
| cg01293179 | *HOXD8* |
| cg10111328 |  |
| cg10536898 |  |
| cg22660542 | *HOXC8* |
| cg26537478 | *MEIS1* |
| cg16324018 | *ELMOD3;RETSAT* |
| cg19177125 | *GATA6* |
| cg01593673 | *HOXB3* |
| cg16197388 | *PSG3* |
| cg25941985 |  |
| cg19506686 |  |
| cg01519253 |  |
